# GLP-1 RA for cardiometabolic risk reduction in obesity – How do we best describe benefit and value?

**DOI:** 10.1016/j.ajpc.2024.100682

**Published:** 2024-05-18

**Authors:** Sant Kumar, Michael J. Blaha

**Affiliations:** a MedStar Georgetown University Hospital, Washington, D.C. 20007, United States; b Johns Hopkins Ciccarone Center for the Prevention of Cardiovascular Disease, Baltimore, MD 21287, United States

**Keywords:** GLP-1 RA, Semaglutide, Weight management, obesity, cost effectiveness

## Abstract

How do we assess the overall benefit and value of GLP1-RAs? Current clinical trials often focus narrowly on individual atherosclerotic cardiovascular endpoints like MACE, potentially missing broader GLP-1 RA benefits across multiple comorbidities. Herein, we set out a framework for expanding outcome analyses in large trials that we believe will provide a more holistic understanding of GLP-1 RA benefits across the cardio-kidney-metabolic (CKM) spectrum, guiding patient care, guidelines, and insurance coverage decisions.

Glucagon-like peptide 1 receptor agonists (GLP-1 RA) are incretin analogs. They reduce glucose levels after meals and promote weight loss by reducing appetite and delaying gastric emptying [1,2]. In 2021 the Food and Drug Administration (FDA) approved semaglutide, a GLP-1 RA, to be used as a once-weekly injection for chronic weight management in adults who are obese or overweight (BMI >27 kg/m^2^) and have at least one weight-related condition, such as high blood pressure, type 2 diabetes, or high cholesterol [3]. Due to its promotion of weight loss, semaglutide has become very popular and has even led to supply shortages [4].

The increasing availability and cost of certain drugs are causing concerns about fairness and affordability. Health insurance may not cover medications like semaglutide when used exclusively for obesity, which means patients may have to pay up to $1000 per month out of their pocket [5]. Since 2003, the Centers for Medicare & Medicaid Services has not included weight-loss medications in Medicare insurance coverage [5]. Therefore, it is crucial to evaluate the benefits and cost-effectiveness of such medications to ensure appropriate coverage. This evaluation is particularly important in 2024 as GLP-1 RAs appear to have substantial additional benefits beyond weight loss.

Indeed, there is growing evidence that supports the interplay between obesity, diabetes, chronic kidney disease (CKD), and cardiovascular disease (CVD) [6]. Nearly half of all patients with heart failure (HF) have CKD [7], and diabetes and obesity serve as predictors of cardiovascular mortality among patients with CVD and HF [8,9]. Over the past decade, several studies have demonstrated that GLP-1 RA benefits go beyond weight loss and extend across the cardiovascular-kidney-metabolic (CKM) syndrome.

The Semaglutide in Patients with Heart Failure with Preserved Ejection Fraction and Obesity (STEP-HFpEF) trial compared the use of semaglutide to placebo in patients with heart failure with preserved ejection fraction (HFpEF) and body mass index of 30 or higher [10]. A total of 529 patients participated in the trial, and the results showed that in patients with HFPEF and obesity, semaglutide treatment reduced symptoms and physical limitations, improved exercise function, and reduced body weight [10]. These results have now been replicated in the type 2 diabetes population [11].

Two meta-analyses of several cardiovascular outcome trials explored whether the benefits of GLP-1 RA extend beyond MACE in patients with type 2 diabetes [12,13]. This included a composite kidney outcome comprising the development of macroalbuminuria, doubling of serum creatinine, or at least a 40 % decline in estimated glomerular filtration rate (eGFR), kidney replacement therapy, or death due to kidney disease. Both meta-analyses had over 50,000 patients and showed a significant 21 % and 17 % reduction in the composite kidney outcome, respectively [12,13]. Recently, it was announced that the FLOW trial of semaglutide in patients with type 2 diabetes and chronic kidney disease would be stopped early for benefit on important renal outcomes [14].

Most importantly, semaglutide was recently studied in a multicenter, double-blind, randomized, placebo-controlled, event-driven superiority cardiovascular outcomes trial called SELECT (Semaglutide and Cardiovascular Outcomes in Obesity without Diabetes) [15]. The SELECT trial demonstrated that weekly semaglutide was more effective than placebo in reducing MACE (major adverse cardiovascular events) in patients who have preexisting atherosclerotic cardiovascular disease, are overweight or obese, but do not have diabetes [15]. Moreover, patients who were treated with once-weekly subcutaneous semaglutide showed a significant decrease in key secondary endpoints of all-cause mortality, hospitalization due to heart failure, and a composite nephropathy endpoint [15].

It is important to note that no clinical trial has yet been designed to examine the *total* benefits of GLP-1 RA medications on a composite of CKM outcomes like MACE, progression of chronic kidney disease (CKD), reducing heart failure, and/or improving quality of life. Therefore, focusing large clinical trials only on a single potential benefit of GLP-1 RA (such as reducing MACE in SELECT) may not capture the extent to which these medications may be cost-effective in reducing the multiple intertwined comorbidities of CKM syndrome. This may have distinct implications for consideration of insurance coverage.

To date, essentially all clinical trials are designed to study a discrete time-to-first event endpoint, such as overall survival free of or time to a cardiovascular event like MACE [16]. This makes sense for drugs that are expected to have a relative narrow mechanism of action on a narrow set of outcomes. An example of this was the trial, Further Cardiovascular Outcomes Research With PCSK9 Inhibition in Subjects With Elevated Risk (FOURIER) [17]. FOURIER demonstrated that the addition of evolocumab to statin therapy significantly reduced atherosclerotic cardiovascular events (ASCVD) in high-risk secondary prevention patients compared to placebo. As expected, evolocumab has not shown a benefit in patients with chronic kidney disease and/or heart failure, unlike semaglutide. There has been no suggestion of reduced all-cause mortality [17,18]. A cost-effectiveness analysis focused on the narrow ASCVD benefits found that evolocumab is still more expensive than the commonly accepted willingness-to-pay threshold [19,20]. It is important to note that insurance providers may only cover the cost of evolocumab for patients with ASCVD who have tried high intensity statins for 8 weeks and still have low density lipoprotein cholesterol levels of 70 mg/dL or higher. Alternatively, patients with a diagnosis of familial hypercholesterolemia may also be eligible for coverage [21]. This information is based on clinical trial results such as those from FOURIER. Considering a wider range of outcomes would not affect the decision to use evolocumab, as the impact on reducing all-cause mortality still remains highly uncertain. However, focusing only on ASCVD or any individual outcome may significantly underestimate the cost-effectiveness of semaglutide, as it has been proven to improve outcomes in patients with ASVCD, CKD, HF, and all-cause mortality.

The FDA has issued guidelines on how to interpret results from clinical trials that involve multiple endpoints. However, there is a concern that analyzing multiple endpoints in a single trial could lead to incorrect conclusions about the effects of the drug on one or more endpoints if the study is not appropriately adjusted for multiplicity [22]. Despite this concern, the emergence of GLP-1 RA medications and their potential benefits on various linked disease processes necessitate the expansion of time-to-first event endpoints to include broad “total benefit” outcomes, including MACE, CKD progression, and HF. Also, commonly observed “event cascades” must be considered. In the case of GLP-1 RA, secondary analysis of these “total benefit” outcomes could involve aspects related to the progression of CKM within an individual patients, for example from the onset of renal disease to heart failure, a first MACE leading to incident kidney disease, or onset of heart failure and subsequent readmissions.

A small step forward in the approach to exploring the benefits of these incretin-based therapies is being implemented in a study called SURMOUNT-MMO (A Study of Tirzepatide on the Reduction on Morbidity and Mortality in Adults With Obesity) [23].^(p. 3)^ The primary outcome of the study is time to the first occurrence of any event of a broad combined outcome. This combined outcome consists of all-cause death, non-fatal myocardial infarction, non-fatal stroke, coronary revascularization, or heart failure events [23].^(p. 3)^ Although SURMOUNT-MMO is a promising development for capturing the total benefits of GLP-1 RA, more innovative approaches are necessary to measure the total effects of these medications on patients with CKM syndrome.

As such, it is important to review the design and analysis of large outcomes trials in the CKM space and expand our understanding of overall benefit. For therapies such as GLP-1 RA, we suggest organizing outcomes based on the degree of multi-organ benefit, instead of classifying individual outcomes as primary and secondary outcomes given their benefits extend beyond a single organ system or overall mortality. One possibility is to consider clinical outcomes in a new way. For example, we suggest combining MACE and HF for the first outcome, called "Total Cardiovascular Disease." When available, atrial fibrillation could also be included in this outcome. The next outcome would include MACE, HF, and CKD progression, to be called "Total CKM Events." In certain settings, obstructive sleep apnea could be included in this outcome, or as the field evolves, potentially metabolic dysfunction-associated steatotic liver disease (MASLD). Lastly, a comprehensive endpoint composite related to obesity could include MACE, HF, CKD progression, total hospitalizations, and all-cause mortality and be called “Total Benefit.” Additional analyses should examine event cascades and recurrent events as well as incident multi-morbidity (emergence of several component outcomes in an individual patient at the same time). Additionally, we suggest pre-specified secondary (not exploratory) outcomes to include patient-reported quality of life and key musculoskeletal outcomes. GLP-1 receptor agonists have displayed potential in these areas in separate studies, with potentially profound impacts on cost-effectiveness from a societal point-of-view (Fig. 1).

**Fig. 1:**
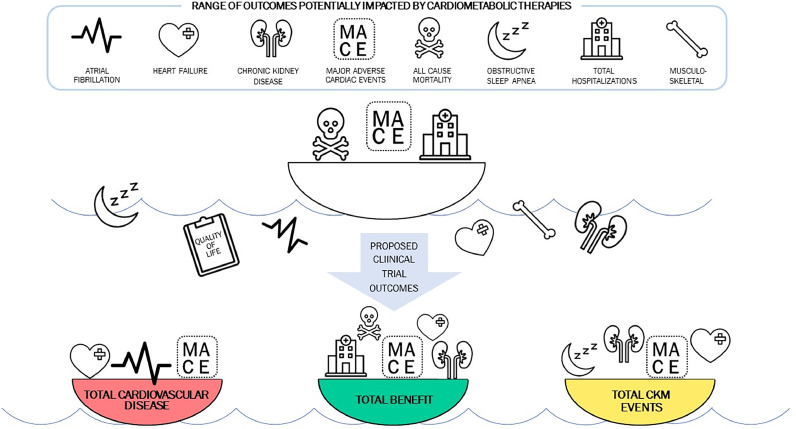
Given potential impact on many organ systems, for comprehensive assessment of cardiometabolic medication benefit, we propose a new schema. For example, we propose merging major adverse cardiac events (MACE) and heart failure (HF) and potentially atrial fibrillation into "total cardiovascular disease" (Bottom Left, Red). "Total CKM Events" would include total cardiovascular disease and add chronic kidney disease (CKD) progression and potentially obstructive sleep apnea (Bottom Right, Yellow). A comprehensive "Total Benefit" endpoint for a cardiometabolic therapy would integrate total cardiovascular and CKM events as well as all-cause mortality and total hospitalizations (Middle, Green). Secondary analysis of the key outcomes should address event cascades and recurrent events as well as incident multi-morbidity. Prespecified secondary outcomes should focus on crucially important patient-reported quality of life and key musculoskeletal outcomes, rather than relegate these to exploratory analysis. Fig. 1. Integrated Endpoint Framework for Assessment of Efficacy of a Cardiometabolic Therapy.

While sacrificing some precision on disease-specific mechanisms, we believe that integrating these outcomes analyses from large-scale trials will better capture the total benefits of medications in the CKM space and directly inform patient decision-making, guidelines, and payer decisions. This will also help us to identify particular patient groups who can receive the most net benefit from these medications, for example, a patient who has obesity, subclinical coronary artery disease, diastolic dysfunction, and limited quality of life due to musculoskeletal pain or disability. Identifying these individuals would help ensure that common sense insurance coverage is provided to those who need the medication the most.

In conclusion, the development of GLP-1 RA, exemplified by semaglutide, represents a promising breakthrough, shifting thinking around what defines a positive “cardiovascular outcomes trial”. However, the increasing popularity of semaglutide due to its weight loss and cardiovascular benefits raises concerns about affordability and insurance coverage, with potential financial burdens on patients. Indeed, pharmaceutical companies must also take on the responsibility of managing the costs of their drugs to ensure affordability. In fact, in early 2024, it seems like access to these medications has actually decreased despite new data. As we navigate the evolving landscape of cardiometabolic disease management, it becomes imperative to think broadly about calculation of cost-effectiveness to include broader individual and societal impact on key outcomes *across the CKM continuum*, such as kidney function and quality of life in conditions like HFpEF. By broadening our understanding of GLP-1 RA medications beyond MACE, we can better inform future trial designs and pave the way for a more holistic approach to cardiovascular care, ensuring that the benefits of these medications reach a wider population while maintaining cost-effectiveness and attention to value. While semaglutide is reducing waistlines, paradoxically, it is making wallets heavier to carry, suggesting that our pockets need to be as deep as our understanding of its benefits to truly balance the scales of health and economy.

## Author contributions

SK designed the study and wrote the paper. MJB designed the study and supervised all aspects of this paper, from its conception to the manuscript's creation.

## Funding

None.

## Disclaimer

The views expressed here are those of the authors.

## CRediT authorship contribution statement

**Sant Kumar:** Writing – review & editing, Writing – original draft, Visualization, Methodology, Investigation. **Michael J. Blaha:** Writing – review & editing, Supervision, Resources, Project administration, Methodology, Investigation, Conceptualization.

## Declaration of competing interest

The authors declare that they have no known competing financial interests or personal relationships that could have appeared to influence the work reported in this paper.
